# Telemonitoring and/or self-monitoring of blood pressure in hypertension (TASMINH4): protocol for a randomised controlled trial

**DOI:** 10.1186/s12872-017-0494-5

**Published:** 2017-02-13

**Authors:** Marloes Franssen, Andrew Farmer, Sabrina Grant, Sheila Greenfield, Carl Heneghan, Richard Hobbs, James Hodgkinson, Susan Jowett, Jonathan Mant, Una Martin, Siobhan Milner, Mark Monahan, Emma Ogburn, Rafael Perera-Salazar, Claire Schwartz, Ly-Mee Yu, Richard J. McManus

**Affiliations:** 1 Nuffield Department of Primary Care, Oxford University, Oxford, UK; 2 Primary Care Clinical Sciences, University of Birmingham, Birmingham, UK; 3 Department of Public Health and Primary Care, University of Cambridge, Cambridge, UK

**Keywords:** Hypertension, Self-monitoring, Telemonitoring, Primary care

## Abstract

**Background:**

Self-monitoring of hypertension is associated with lower systolic blood pressure (SBP). However, evidence for the use of self-monitoring to titrate antihypertensive medication by physicians is equivocal. Furthermore, there is some evidence for the efficacy of telemonitoring in the management of hypertension but it is not clear what this adds over and above self-monitoring. This trial aims to evaluate whether GP led antihypertensive titration using self-monitoring results in lower SBP compared to usual care and whether telemonitoring adds anything to self-monitoring alone.

**Methods/Design:**

This will be a pragmatic primary care based, unblinded, randomised controlled trial of self-monitoring of BP with or without telemonitoring compared to usual care. Eligible patients will have poorly controlled hypertension (>140/90 mmHg) and will be recruited from primary care. Participants will be individually randomised to either usual care, self-monitoring alone, or self-monitoring with telemonitoring. The primary outcome of the trial will be difference in clinic SBP between intervention and control groups at 12 months adjusted for baseline SBP, gender, BP target and practice. At least 1110 patients will be sufficient to detect a difference in SBP between self-monitoring with or without telemonitoring and usual care of 5 mmHg with 90% power with an adjusted alpha of 0.017 (2-sided) to adjust for all three pairwise comparisons. Other outcomes will include adherence of anti-hypertensive medication, lifestyle behaviours, health-related quality of life, and adverse events. An economic analysis will consider both within trial costs and a model extrapolating the results thereafter. A qualitative sub study will gain insights into the views, experiences and decision making processes of patients and health care professionals focusing on the acceptability of self-monitoring and telemonitoring in the routine management of hypertension.

**Discussion:**

The results of the trial will be directly applicable to primary care in the UK. If successful, self-monitoring of BP in people with hypertension would be applicable to hundreds of thousands of individuals in the UK.

**Trial registration:**

ISRCTN 83571366. Registered 17 July 2014

## Background

Blood pressure (BP) is a key risk factor for cardiovascular disease, the largest cause of morbidity and mortality worldwide [1, 2]. A 10 mmHg reduction in BP is estimated to lead to a 41% reduction in stroke and a 22% reduction in coronary heart disease [3]. National and international surveys suggest that, despite significant improvements in recent years, BP control within the population remains sub-optimal [4–7]. Self-monitoring as an intervention has been shown to reduce BP [8, 9], improve adherence to antihypertensive medication [10], and reduce primary care consultation rates at no additional cost [11]. Telemonitoring is associated with reduced BP but there are limited data relevant to a UK context, particularly with longer than 6 months follow-up [12, 13]. Furthermore, the evidence concerning the use of self-monitoring to guide GPs to titrate anti-hypertensives is equivocal with one systematic review finding the effect of self-monitoring on BP reduction becomes non-significant when a medication titration protocol is used [14].

The current (2004, updated 2006 & 2011) National Institute for Health and Care Excellence (NICE) guideline states: “the value of routinely using … home (BP) monitoring devices has not been established: their appropriate use in primary care remains an issue for further research” [15]. This trial aims to evaluate whether GP led antihypertensive titration using self-monitoring results in lower systolic BP (SBP) compared to usual care and whether telemonitoring reduces BP over and above self-monitoring alone.

## Methods and design

### Study aims, research questions and outcomes

The primary aim of TASMINH4 is to compare the management of hypertension in primary care using self-monitored BP to make treatment decisions, with or without telemonitoring, with usual care.

The trial has four main research questions:

1. Does self-monitoring guided titration of antihypertensive medication, with or without telemonitoring, lead to better BP control in people with hypertension in primary care?
2. Does self-monitoring guided titration of antihypertensive medication with telemonitoring lead to better BP control than self-monitoring guided titration using paper-based recording of BP?
3. Is the management of hypertension through self-monitoring cost-effective?
4. What are the views of patients and their clinicians regarding self-monitoring guided titration of antihypertensive medication, with or without telemonitoring?

The primary outcome of the trial will be the difference in SBP (mean of 2^nd^/3^rd^ readings, mmHg) at 12 month follow-up between intervention and control adjusted for baseline BP, gender, BP target, CVD history and practice.

Secondary outcomes (see Table 1, in each case also adjusted for baseline values and co-variates) will include:

**Table 1:** Data collection throughout the trial

Baseline only:
1. Demographic questions: including age, race, marital status, occupation, and education
2. Duration of hypertension [from notes]
3. Past medical history [from notes and corroborated by patients]
4. Contraindications to anti-hypertensives
5. Short orientation memory test [22]
6. Height
Baseline and follow-up:
1. New medical history (in last 6/12 months)
2. Blood pressure (sitting plus standing)
3. Current anti-hypertensive medications including complementary herbal or dietary supplements for BP lowering
4. Weight and waist circumference
5. Symptoms part plus short form of Illness Perception Questionnaire [23]
6. Short-form of the State-Trait Anxiety Inventory [24]
7. EQ-5D 5L [25]
8. BP measurement preference [26] [Baseline and 12 m follow up only]
9. Medication Adherence Rating Scale (MARS) Questionnaire [27] [Baseline and 12 m follow up only]
10. Beliefs about Medicines Questionnaire [28] [Baseline and 12 m follow up only]
11. Stanford Expectations of Treatment Scale (SETS) Items 1–6 [29] [Baseline and 12 m follow up only]
12. Lifestyle questions: alcohol (Audit-C [30]), diet (Short Food Frequency Questionnaire [31]), exercise (Godin Leisure-Time Exercise Questionnaire [32]), smoking (Smoking tool kit [33]) [Baseline and 12 m follow up only]

Blood Pressure Outcomes

- Difference in SBP at 6 months follow up between intervention and control
- Difference in diastolic BP at six and 12 months follow up between intervention and control
- All BP comparisons using mean of 2^nd^-6^th^ readings (BP-Tru automated blood pressure monitor, (BP TRU BPM 200; BP TRU Medical Devices, Coquitlam, BC, Canada) [16].

Tertiary Outcomes

Adverse events

- Clinical Events: admissions, cardiovascular events, deaths
- Anxiety
- Side effects of medication

Medication Outcomes

- Medication prescription: both number and defined daily dose
- Adherence to medication
- Beliefs about medicines, expectations of treatment, illness perceptions

Fidelity to intervention

- GP fidelity to medication titration protocol
- Patient fidelity to monitoring regime

Lifestyle

- Alcohol, diet, smoking, exercise

Quality of Life

- EQ-5D-5L

Economic Outcomes:

- Resource use and costs
- Within trial effectiveness will be assessed in terms of cost per 1 mmHg blood pressure reduction. Long term cost effectiveness will be assessed by linking differences in blood pressure observed in the trial to cardiovascular events with a lifetime horizon.

Qualitative Analysis:

The views of patients and clinicians will be assessed through depth interviews in the qualitative sub study (see below for further details).

### Study design and setting

TASMINH4 is a pragmatic unblinded individual patient randomised controlled trial with automated ascertainment of outcome and embedded economic and qualitative analyses.

### Study population

The study population will comprise people with poorly controlled hypertension managed in primary care. Eligibility criteria will be: age over 35 years, on the hypertension register, not already taking more than 3 anti-hypertensive agents, BP above 140/90 mmHg at the baseline clinic, and on a stable dose of current antihypertensive medication for at least 4 weeks prior to trial entry. Exclusion criteria will be orthostatic hypotension (20 mmHg or more systolic drop after standing for one minute, in order to avoid adverse events), BP not managed by their GP (limited possibility of antihypertensive titration), diagnosed atrial fibrillation (automated monitors not validated), unwilling to self-monitor, dementia or score over 10 on the short orientation memory concentration test (inability to undertake self-monitoring), female participant who is pregnant, lactating or planning pregnancy during the trial (management of essential hypertension in pregnancy is different), the partner or spouse of an individual already randomised in the trial (to avoid clustering within families), Chronic Kidney Disease (CKD) grade four or worse, any grade of CKD with proteinuria (both may have different BP targets), participants who have participated in another research trial involving antihypertensive medication in the past 4 weeks.

Potentially eligible patients will be identified from general practices via the UK Clinical Research Network (CRN). GP surgery staff supported by the research team/ CRN will conduct a practice-based computer search to identify patients that fulfil the eligibility criteria. GPs will be asked to check these computer generated lists and to remove people who are known to have terminal illnesses, those not managed by the GP and those thought to be unsuitable for the study in the opinion of the GP. The remainder will receive a postal invitation with a reminder after two weeks. Those wishing to decline participation may voluntarily return a form detailing basic demographic details as well as their reasons for declining.

### Baseline clinics

Patients will attend an initial baseline clinic where the study will be explained, informed consent gained, clinical measurements (height, weight, and BP) taken, demographics, past medical history, and key health behavioural and attitudinal related data collected (Table 1) by a trained researcher (either a research nurse, trained practice nurse, or member of the research team). Measurement of BP will use a validated automated electronic sphygmomanometer (BP TRU BPM 200). After five minutes of rest, six seated BP readings will be taken at 1-min intervals, of which the mean of the 2^nd^ and 3^rd^ reading will comprise the primary outcome. Randomisation will take place at the end of the baseline clinic.

### Randomisation

Patients will be randomised into one of three groups: self-monitoring alone, self-monitoring and telemonitoring, or usual care (1:1:1), using a fully-validated internet based randomisation system with manual telephone-based back up. Randomisation will be stratified by practice and minimisation, with a non-deterministic algorithm, will be used to ensure balance in baseline BP, gender and BP target (standard, older person or diabetic) across the groups.

### Intervention and control groups

***Usual care*** will consist of the participant’s BP being measured by their GP and/ or nurse at their practice, and adjustment of medication based on these measurements at the discretion of the health care professional.

The participants in the ***self-monitoring*** groups (self-monitoring alone and self-monitoring with telemonitoring) will be trained to monitor their BP using an automated electronic sphygmomanometer (Omron M10-IT) [17]. Patients will self-monitor BP daily for the first week of each month of the study. They will take their BP twice in the morning and evening (i.e. four times in all per day) [15]. Participant training will include instructions as to what to do in the presence of a high or low reading, using a guideline that contains simple colour-coded instructions. Very high or very low readings that persist when a third reading is taken five minutes after the second reading will trigger the patient to contact their practice for further advice.

### Medication reviews

All participants will be asked to book an appointment approximately one week later with the GP or practice nurse for a medication review. For the patients in the usual care arm, the GP will review the medication based on BP measurements taken in the clinic. The patients in the self-monitoring arms will monitor their BP for a week and bring the readings to their appointment. The GP will base the medication review on the readings done by the patient at home. After the medication review the GP/ nurse will register the patients in the telemonitoring arm on the text system and will train the patient in using the system.

### Communication of home readings

#### Self-monitoring group

Participants in the self-monitoring group will complete a simple two-part carbon copy form each month to record their daily BP readings, one copy to be kept by the patient, and one posted to the practice in a reply-paid envelope. The GP/ nurse will be asked to review these BP readings each month to determine whether a change in medication is required and the GP/ nurse will contact the patient if a medication change is required. At follow-up (6 and 12 months), data from participants’ BP machines will be uploaded and sent to the research team.

#### Telemonitoring group

Participants will be trained in the use of the telemonitoring equipment before commencing. Participants in the telemonitoring group will send their readings to a secure centralised database using a free SMS text message with web-based data entry back up. They will receive a reminder the day before their week of measurements, and two additional reminders in the week if no measurements are received by the system. Mean BP will be calculated automatically. High or low readings will trigger text alerts to the patient to contact their surgery for a BP check. The GP/ nurse will review the readings on a monthly basis via a web-based interface to determine whether a change in medication is required and the GP/nurse will contact the patient if a medication change is required. At follow-up (6 and 12 months), data from participants’ BP machines will be uploaded and sent to the research team.

### Target blood pressure

Target BP will be based on the NICE hypertension guideline with adjustment downwards by 5/5 mmHg for home as compared to office readings [18]. Thus people without diabetes under 80 years will have a home target of ≤135/85 mmHg; people over 80 years will have a home target of ≤145/85 mmHg; finally, people with diabetes will have a home target of ≤135/75 mmHg [15]. People requiring lower than standard targets will be excluded (hence the exclusions for CKD4 and proteinuria) (see Table 2).

**Table 2:** Blood Pressure targets for the different groups for home readings and for readings taken in clinic

	BP target (mmHg)	BP target (mmHg)
Readings	Taken at home	Taken in clinic
<80 years	≤135/85	≤140/90
>80 years	≤145/85	≤150/90
Diabetic	≤135/75	≤140/80

### Follow-up

**Fig. 1:**
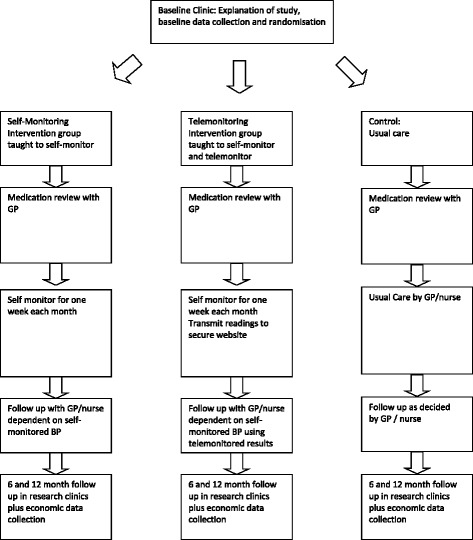
Flow through the trial

All patients will be asked to attend two follow-up clinics; 1 at 6 months and 1 at 12 months. Each clinic will be timetabled for no more than an hour, during which patients will have their BP and weight measured by the research team and will be asked to complete a questionnaire similar to the one completed at baseline. Participant flow through the trial is shown in Fig. 1. All data is collected on a case report form especially designed for the study.

Patients who withdraw will not be replaced, but asked if they are prepared to attend follow-up clinics.

### Sample size considerations

The study requires a total sample of 1110 patients to be recruited with 370 patients in each arm. This is based on an assumption of a common standard deviation of 17 mmHg and a three way pairwise comparison. At least 367 participants per group (allowing for 15% attrition) would allow detection of a 5 mmHg difference between the groups (i.e. standardised effect size = 0.3) with 90% power and an adjusted alpha of 0.017 (2-sided) to account for all three pairwise comparisons. We estimate that around 120–150 practices will be required to recruit this size of sample, assuming an average list size of 7000, with a prevalence of hypertension of 13%, of whom 16% will respond to a trial invitation and 40% of these will be eligible. This corresponds to around 7–10 patients per practice of 7000 patients.

### Statistical analysis

Analysis will be on an intention-to-treat basis. A mixed model will analyse the primary outcome utilising data collected at 6 and 12 months from randomisation, adjusting for baseline BP measure and minimisation variables. An advantage of the mixed model is that it implicitly accounts for data missing at random. The model will include a random intercept for each participant to account for the repeated measures on the same participant. Time and randomised group will be fitted as fixed effects, with minimisation variables, region and history of CVD fitted as covariates. An interaction term between time and randomisation group will be included so that possible differences of treatment effect can be assessed at each time point. Similar methods will be used for other continuous outcomes. The two self-monitoring groups will first be compared to the usual care group. If both treatments are found to be more effective than usual care, they will be compared to each other.

Sensitivity analyses will be carried out to examine the robustness of the results with different assumptions about departures from randomisation policies and handling of missing data. A detailed statistical analysis plan will be prepared before any analyses are undertaken and before the trial database is locked.

### Data management

Double data entry will be employed for all trial paperwork. The second data entry person will resolve differences between first and second pass (differences will be identified at point of submission by the data entry module within OpenClinica).

### Potential risks

It is anticipated that the potential risks of this study are low and similar to those attributable to usual care. A particular issue is the possibility of increased anxiety where a patient finds an excessively high or low reading. The patient guideline will advise contact with the supervising physician or nurse in the case of excessively high or low readings. Training of participants will cover repeated measurements in the case of unusually high or low readings and a study “Freephone” helpline will be available in the guideline. Data on adverse events will be collected.

### Economic sub-study

The economic evaluation will comprise both a within-trial analysis and model-based analysis to extrapolate beyond the trial results.

- *Trial-based analysis*

NHS costs will be determined for health care resource use over the 12 month follow-up period of the trial. Resource use will include primary care consultations (GP and practice nurse visits), secondary care referrals, hospital inpatient stays and antihypertensive medications. Data on trial-specific resources such as consultations, equipment for self-monitoring and telemonitoring and training will also be collected. Unit costs will be derived from published sources.

The cost-effectiveness analysis will consider the cost per additional 1 mmHg reduction in Systolic BP from baseline to 12 months. A cost-utility analysis will determine the cost per Quality-Adjusted Life Year (QALY) gained over the same period, using patient responses to the EQ-5D 5L. The results for both outcomes will be expressed in terms of Incremental Cost-Effectiveness Ratios (ICERs). The base case economic evaluation will adopt an NHS perspective. The analysis will also consider all three arms of the trial, comparing self-monitoring with and without telemonitoring to usual care.

Sensitivity analysis will test the robustness of the results. Key parameters will be varied to determine the impact of changes on results. Non-parametric bootstrapping and probabilistic sensitivity analysis will be undertaken to explore uncertainty in the confidence to be placed on the results of the economic analysis and cost effectiveness acceptability curves presented.

b) *Model-based analysis*

A Markov model-based analysis with patient level simulation will consider long-term cost effectiveness by linking intermediate outcomes (i.e. change in BP) to cardiovascular events, and will consider the BP monitoring options within the trial and usual care. The model will determine the cost per additional QALY gained for alternative monitoring scenarios.

Data from the trial and literature will inform the probability of these events occurring and the risk reduction afforded by the alternative strategies. Attached to each health state will be associated health state utility values in order that QALYs can be calculated. Quality of life on each treatment strategy will be obtained from the trial data on EQ-5D 5L, and previous studies will inform utility values for cardiovascular disease health states. Costs of monitoring and the therapies prescribed in each strategy and acute and long term costs of cardiovascular events will be obtained within the trial and from the literature.

The base-case will be conducted from a health and personal social services perspective. The model will be run over patient lifetime, with costs and benefits discounted at a rate of 3.5% [19]. In order to explore uncertainties in the analyses, deterministic sensitivity analysis will test the robustness of the model when varying key model parameters and structural assumptions, including assumptions around side effects of treatment and adherence to medication. Probabilistic sensitivity analysis will be undertaken to incorporate the uncertainty around parameter values and quantify the overall decision uncertainty, and inform whether further research is required.

### Qualitative sub-study

The qualitative component of the trial will gain insights into the views, experiences and decision-making processes of patients and health care professionals regarding self-monitoring and telemonitoring in the routine management of hypertension.

It is anticipated that up to 30 patients and up to 30 clinicians who have taken part in the trial will be interviewed. Where carers were involved in the process of hypertension care, they will be invited to join the patient interviews. Participants will be selected purposively from participating practices to ensure a maximum variety sample reflecting the range of professional and participant characteristics [20]. Interviews will take place when participants have had a minimum of 6 months trial participation to ensure current relevance to study.

Consent will be sought from all patients, informal carers/relatives and health care professionals taking part, including for the use of anonymised quotes.

The interviews will focus on identifying perceived barriers and facilitators to implementing either the telemonitoring or paper-based system in routine practice. Decisional processes and information sharing between patient, practitioner and practice staff relating to implementation will be further explored. Interviews will be recorded and transcribed verbatim.

Interviews will be analysed thematically to bring out both ‘articulated’ data (direct responses to questions on the areas described above) as well as ‘emergent’ data (new information which emerges from comparison of themes) [21].

## Discussion

This article describes the protocol for the TASMINH4 study, a randomised controlled trial assessing whether GP led antihypertensive titration using self-monitoring results in lower SBP compared to usual care and whether telemonitoring reduces BP over and above self-monitoring alone.

The results will provide data on the effects of self-monitoring with or without telemonitoring as a means of titrating blood pressure in UK primary care. Secondary outcomes will include a process evaluation and information about potential harms. Linked qualitative and economic work will aid understanding of how these interventions have worked in practice and cost effectiveness.

The results of the trial will be directly applicable to primary care in the UK. If antihypertensive drug titration in primary care using self-monitoring of BP is found to be successful in the management of hypertension, then it would be applicable to many hundreds of thousands of individuals in the UK. Importantly the trial will also inform the use (or not) of telemonitoring alongside self-monitoring of BP, feeding into guidelines in the UK and beyond.

## Abbreviations

BP: Blood pressure
CKD: Chronic kidney disease
CVD: Cardiovascular disease
EQ-5D-5L: European Questionnaire 5 Dimensions
GP: General practitioner
ICERs: Incremental cost-effectiveness ratios
mmHg: Millimetre of mercury
NHS: National Health Service
NICE: National Institute for Health and Care Excellence
QALY: Quality-adjusted life year
SBP: Systolic blood pressure
UK-CRN: United Kingdom clinical research network
